# Fabrication and In Vitro/Vivo Evaluation of Drug Nanocrystals Self-Stabilized Pickering Emulsion for Oral Delivery of Quercetin

**DOI:** 10.3390/pharmaceutics14050897

**Published:** 2022-04-20

**Authors:** Zhe Wang, Bo Dai, Xiaohan Tang, Zhihui Che, Fei Hu, Chengying Shen, Wei Wu, Baode Shen, Hailong Yuan

**Affiliations:** 1 Department of Pharmacy, Air Force Medical Center, PLA, Beijing 100142, China; wangzhe20220325@163.com (Z.W.); daibo0716@163.com (B.D.); cczh0519@163.com (Z.C.); hf19970120@163.com (F.H.); 2 College of Pharmacy, Chengdu University of Traditional Chinese Medicine, Chengdu 611137, China; 3 School of Pharmacy, Anhui Medical University, Hefei 230032, China; z17758133740@163.com; 4 Key Lab of Modern Preparation of Traditional Chinese Medicine, Ministry of Education, Jiangxi University of Chinese Medicine, Nanchang 330004, China; 5 Department of Pharmacy, Jiangxi Provincial People’s Hospital, Nanchang 330004, China; shenchengying0728@163.com; 6 Key Laboratory of Smart Drug Delivery of Ministry of Education, School of Pharmacy, Fudan University, Shanghai 201203, China; wuwei@shmu.edu.cn

**Keywords:** pickering emulsion, self-stabilizing, poorly soluble drug, quercetin, nanocrystals, oral bioavailability

## Abstract

The aim of this study was to develop a new drug nanocrystals self-stabilized Pickering emulsion (NSSPE) for improving oral bioavailability of quercetin (QT). Quercetin nanocrystal (QT–NC) was fabricated by high pressure homogenization method, and QT–NSSPE was then prepared by ultrasound method with QT–NC as solid particle stabilizer and optimized by Box-Behnken design. The optimized QT–NSSPE was characterized by fluorescence microscope (FM), scanning electron micrograph (SEM), X-ray diffraction (XRD), and differential scanning calorimetry (DSC). The stability, in vitro release, and in vivo oral bioavailability of QT–NSSPE were also investigated. Results showed that the droplets of QT–NSSPE with the size of 10.29 ± 0.44 μm exhibited a core-shell structure consisting of a core of oil and a shell of QT–NC. QT–NSSPE has shown a great stability in droplets shape, size, creaming index, zeta potential, and QT content during 30 days storage at 4, 25, and 40 °C. In vitro release studies showed that QT–NSSPE performed a better dissolution behavior (65.88% within 24 h) as compared to QT–NC (50.71%) and QT coarse powder (20.15%). After oral administration, the AUC_0–t_ of QT–NSSPE was increased by 2.76-times and 1.38 times compared with QT coarse powder and QT–NC. It could be concluded that NSSPE is a promising oral delivery system for improving the oral bioavailability of QT.

## 1. Introduction

Quercetin (QT), a plant flavonoid extracted and isolated from *Sophorajaponica* L., was widely present in several edible fruits and vegetables [1], which has a variety of pharmacological effects such as anti-inflammatory, anti-oxidant, anti-hypertensive, and neuroprotection [2,3,4,5]. Recent studies suggested that QT could decrease neuronal oxidative stress by scavenging free radicals, inhibiting xanthine oxidase and nitric oxide synthase, and had an improvement effect on Alzheimer’s disease [6,7,8,9]. Nonetheless, QT exhibits poor dissolution behavior due to its low solubility that is only 0.01 mg·mL^−1^ in water at 25 °C [10], which seriously limited its oral bioavailability. In the past few decades, considerable research efforts have been devoted to enhancing the solubility of QT in water, thus improving its oral bioavailability, such as liposomes [11], self-microemulsion [12], and nanocrystals [13,14,15]). But these methods still have some limitations such as poor stability and low drug loading capacity. In addition, they require numerous surfactants, which might lead to severe ecological pollution and some side effects such as neurotoxic damage, allergy, irritancy, renal damage [16,17,18]. Therefore, more efforts should be made to explore a new dosage form for improving oral absorption of QT.

Pickering emulsions, stabilized by insoluble solid particles without any surfactants, were found in 1900s. Compared with the surfactant emulsions, Pickering emulsions have a stable space barrier formed by solid particles adsorbed on the surface of emulsion droplets, which results in great stability [19]. Moreover, Pickering emulsions also have shown other advantages such as eco-friendliness and lower cost [20,21]. Therefore, Pickering emulsion have attracted plentiful attention in food, cosmetic, and pharmaceutical application [22,23,24]. As an emerging drug delivery system, Pickering emulsions were usually used for facilitating the dermal delivery of drugs, enhancing oral bioavailability of insoluble drugs, and controlling the drug release [25,26,27,28].

However, there are still some problems with Pickering emulsions for drug delivery due to the safety of solid stabilizers. Many solid particles could be adopted for Pickering emulsions stabilization, including silica, cellulose, starch granules, and Mg (OH)_2_ [29,30,31,32]. Among these solid particles, silica has been the most frequently used in the drug delivery systems of Pickering emulsions. Nevertheless, massive use of silica might take some adverse effects, such as neurotoxic damage, cytotoxicity, and renal damage [33,34,35,36]. Furthermore, Pickering emulsions usually have low drug loading capacity owing to the limitation of drug solubility in the oil phase. There is thereby an urgent need to explore new approaches to prepare Pickering emulsions with favorable safety profile and high drug loading capacity.

More recently, drug nanocrystals self-stabilized Pickering emulsion (NSSPE), as a new delivery system of drugs with poor water solubility, was developed to improve the water solubility of insoluble drugs thus enhancing its low oral bioavailability. NSSPE was stabilized by the nanocrystals of insoluble drugs without any additional solid particle stabilizers. It has dual advantages of nanocrystals and Pickering emulsions, and also overcome the side effects caused by solid particles such as silica, which might be a good choice for oral delivery of drugs with poor water solubility. In NSSPE, the insoluble drugs cannot only adsorb on the oil-water interface in the form of nanocrystals to stabilize the Pickering emulsions, but also dissolve in the oil inner phase. Accordingly, NSSPE has a better drug loading capacity as compared with the conventional Pickering emulsions. So far, NSSPE had been applied to the flavonoids puerarin and silybin, results showed that it could significantly increase oral absorption of these two drugs when compared with drug coarse powder [24,37].

The purpose of this study was to developed QT nanocrystal self-stabilized Pickering emulsion (QT–NSSPE) for oral delivery of QT. QT nanocrystal (QT–NC) was fabricated by high pressure homogenization method, and QT–NSSPE was then prepared by ultrasound method with QT–NC as solid particle stabilizer and optimized by Box-Behnken design. The morphology, differential scanning calorimetry (DSC), X-ray powder diffraction (XRD), physical stability, and in vitro drug release of QT–NSSPE were investigated. Finally, a pharmacokinetic study of QT–NSSPE was implemented in rats as compare to QT coarse powder, and nanocrystal.

## 2. Materials and Methods

### 2.1. Materials

Quercetin coarse powder (purity > 95%) was purchased from Shaanxi Ci Yuan Biotechnology Co., Ltd. (Xi’an, China); QT reference standard (purity > 98%) and kaempferol (internal standard, purity > 98%) were purchased from Chengdu Pufei De Biotech Co., Ltd. (Chengdu, China); Labrafac Lipophile WL 1349 was obtained from Beijing Fengli Jingqiu Pharmaceutical Co., Ltd. (Beijing, China). HPLC-grade methanol was purchased from Fisher Scientific (Waltham, MA, USA). Other chemicals were analytical grade.

### 2.2. Animals

Specific pathogen free male Sprague-Dawley rats (180–220 g) were acquired from the Keyu Laboratory Animal Center of Beijing (Beijing, China). They received care in compliance with the Principles of Laboratory Animal Care and the Guide for the Care and Use of Laboratory Animals. The protocol of the study was approved by the Institutional Animal Ethics Committee of Air Force Medical Center, PLA of China (No. 2021-74-PJ01). All rats were housed in an environmentally controlled breeding room (25 ± 2 °C, 60 relative humidity, and 12 h cycle of light and dark).

### 2.3. Preparation and Characterization of Quercetin Nanocrystal

Quercetin nanocrystal (QT–NC) was prepared by high pressure homogenization method. Briefly, QT coarse powder was dispersed in 50 mL pure water using a high shear homogenizer (MT-30K, Hangzhou Miu Instruments Co., Ltd., Hangzhou, China) at 13, 000 rpm for 2 min. And then the dispersion was processed through a high pressure homogenizer (AH100D, ATS Industrial Systems Co., Ltd., Suzhou, China) with ten homogenization cycles at 100 MPa.

The mean size of the QT–NC was measured using a size analyzer (Winner 801, Jinan Winner Particle Instruments Stock Co., Ltd., Jinan, China). The morphology of QT–NC was observed by transmission electron microscopy (TEM). QT–NC was dropped on a 200-mesh copper grid and dried in the air for morphological evaluation by TEM (Tecnai G2F20, FEI, Eindhoven, The Netherlands).

### 2.4. Preparation of QT–NSSPE

**Figure 1:**
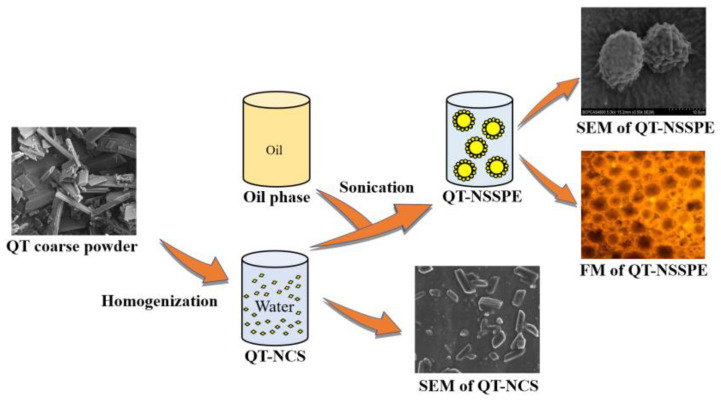
Schematic diagram of formation process of QT–NSSPE.

According to our previous study, Labrafac Lipophile WL 1349 was used as oil phase of QT–NSSPE [38]. The schematic diagram of formation process of QT–NSSPE can be seen in Figure 1. Firstly, the appropriate volume of oil and freshly obtained QT–NC were added into a glass vial to get 10 mL of mixed liquid. Next, the glass vial of mixture was placed into a beaker with ice water and then processed by an ultrasonic cell grinder (JY92-IIN, Ningbo Scientz Biotechnology Co., Ltd., Ningbo, China) with a dipping probe close to the oil/water interface to form QT–NSSPE, for 9 min at an intensity level 6 and 50% pulses. In addition, the duration of ultrasonication was 3 s per time and the interval time was 4 s, in support of heat dissipation.

### 2.5. Experimental Design

The preliminary experiment showed that the oil volume fractions (X_1_), QT concentration (X_2_), and pH (X_3_) had significant effects on the droplets size and drug loading of QT–NSSPE. Therefore, the three factors were chosen as independent variables for a 3-factor, 3-level Box-Behnken design to optimize the formulation of QT–NSSPE. The second-order polynomial models and quadratic response surfaces were generated by Design Expert software. The independent and dependent variables were listed in Table 1, along with their low, middle, and high levels.

### 2.6. Droplets Shape, Droplets Size and QT Content in QT–NSSPE Determination

The droplets shape of QT–NSSPE was observed by microscopy (BX60, Integrated DS-SMC-UI digital imaging system, Olympus Co., Ltd., Tokyo, Japan) and the mean size of the QT–NSSPE droplets were calculated by the software of Image Pro Plus 6.0 (USA). QT–NSSPE of 1 mL was placed into 100 mL of methanol and sonicated for 10 min to make the QT completely dissolve in methanol. After centrifuging at 1000 rpm for 10 min, 1 mL of supernatant was filtered through 0.22-μm millipore filter. The amount of dissolved QT in methanol was determined by HPLC. HPLC analysis was performed on a LC-20A HPLC system (Shimadzu, Tokyo, Japan). Analysis was performed on Diamonsil-C18 column (250 × 4.6 mm, 5 μm, Shimadzu, Tokyo, Japan) with column temperature maintained at 30 °C. The mobile phase consisted of methanol and 0.1% phosphoric acid (59:41, *v*/*v*). The flow-rate was 1 mL·min^−1^ and the detection wavelength was 360 nm (Han et al., 2020). Test of each sample was carried out three times and the results were recorded as an average.

### 2.7. Zeta Potential and Creaming Index

Zeta potential of QT–NSSPE was determined by a Zetasizer (NICOMP 380ZLS, PSS, California, USA) after the emulsion was diluted 20-fold with pure water. The appearance of QT–NSSPE was observed and creaming index (CI) was calculated as follows [19]:

CI = (H_t_/H_0_) × 100% (1)

where H_t_ and H_0_ are the height of the emulsion layer at a certain time and the total height of samples, respectively.

### 2.8. Characteristic of QT–NSSPE

#### 2.8.1. Fluorescence Microscope (FM)

QT has a strong fluorescence when excited by UV-lights [39], therefore, QT–NSSPE was observed by FM to confirm the adsorption behavior of QT–NC on the surface of emulsion droplets. QT–NSSPE was dropped on a glass and observed with a fluorescence microscopy (BX60, Integrated DS-SMC-UI digital imaging system, Olympus Co., Ltd., Tokyo, Japan) immediately.

#### 2.8.2. Scanning Electron Microscope (SEM)

Fresh QT–NSSPE 1 mL was diluted and dropped onto a clean tin foil and then dried at room temperature. The morphology of QT–NSSPE was observed by SEM (S-4800, Hitachi, Tokyo, Japan) after being gold coated in a vacuum by a sputter coater before analysis. At the same time, QT coarse powder and QT–NC were also observed as a control.

#### 2.8.3. Differential Scanning Calorimetry (DSC) and X-ray Powder Diffraction (XRD)

The thermal properties and crystalline nature of QT coarse powder, QT–NC lyophilized powder, and QT–NC adsorbed on the surface of QT–NSSPE droplets were examined by DSC and XRD, respectively. QT–NC was lyophilized by a freeze dryer (Lab-1A-50, Beijing Bo Yikang Experimental Instrument Co., Ltd., Beijing, China) to obtain a dry sample. The QT–NC adsorbed on the surface of QT–NSSPE droplets was collected after centrifugation at 15,000 rpm for 30 min and dried by a freeze dryer. DSC measurements were performed using a thermal analyzer (204 A/G, Netzsch, Bühl, Germany). The samples were analyzed in open aluminum pans and heated at the speed of 10 °C/min from 50 to 350 °C. The X-ray diffractograms were recorded by powder X-ray diffractometry (D/Max-2500PC, Rigaku, Japan) with a CuKalfa radiation (λ = 1.5416 Å). The X-ray diffractograms were performed in a step scan mode with a current of 25 mA and a voltage of 40 kV over the angle range of 7° to 55° at a speed of 1 °/min.

### 2.9. Stability of QT–NSSPE

Fresh QT–NSSPE were stored vertically in tubes at 4, 25, and 40 °C, respectively. The droplets shape, droplets size, CI, zeta potential, and QT content of QT–NSSPE were determined after storage for 5, 10, 15, 20, 25, and 30 days to evaluate the physical stability of QT–NSSPE using the same methods as described in Section 2.6 and Section 2.7.

### 2.10. In Vitro Release Study

Dissolution experiment was performed by dialysis bag diffusion method employing Chinese Pharmacopeia Method II dissolution apparatus (ZRS-8G, Tianda Tianfa Co., Ltd., Tianjin, China). QT–NSSPE, QT–NC, and QT coarse powder suspension (QT–CPS) containing 16.2 mg QT were placed in dialysis bags (sigma) with a molecular weight cutoff of 8–14 kDa, respectively. Then, the dialysis bag was tied and immersed into 900 mL of phosphate buffer saline (PBS, pH = 7.4) containing SDS (1%, *w*/*v*) [40] at 37 °C with a paddle speed of 100 rpm. Samples (1 mL) was withdrawn at 0.5, 1, 2, 4, 6, 8, 12, and 24 h, and immediately replenished with equal volume of the release medium. QT concentration of each sample was determined by HPLC as described in Section 2.6.

In order to determine kinetics and mechanism of drug release, zero-order model, first-order model, Higuchi’s model, and the Korsmeyer-Peppas release model were applied to the in vitro release profile data according to their equations as follows:

M_t_/M_∞_ = k_0_t (2)

M_t_ = M_∞_(1 − e^−kt^) (3)

Higuchi model: M_t_/M_∞_ = k_h_t^1/2^ (4)

Korsmeyer-Peppas model: M_t_/M_∞_ = k_p_t^n^ (5)

where M_t_ is the cumulative amount of drug released at time point t, M_∞_ is the initial amount of drug, t represents time, k_0_, k, k_h_, k_p_ represent the zero-order model, first-order model, Higuchi, and Korsmeyer-Peppas rate constant, respectively, and n represents release/diffusion exponent.

### 2.11. Pharmacokinetic Study in Rats

All animals were fasted overnight with water accessible prior to the experiment. A total of 18 rats were randomly divided into three groups. QT coarse powder suspension (QT–CPS), QT–NC, and QT–NSSPE were intragastrically administered at a dose of QT equivalent to 50 mg·kg^−1^, respectively. To obtain QT–CPS, QT coarse powder was added into pure water and vortexed for 3 min. At predetermined intervals of 0.083, 0.25, 0.5, 1, 2, 4, 6, 8, 12, and 24 h after drug administration, blood samples of about 0.5 mL were collected via retro-orbital venous plexus and placed into pre-heparinized centrifuge tubes. The blood sample was immediately centrifuged at 5000 rpm for 10 min. A 200 μL of the supernatant was gathered and stored at –20 °C until analysis.

A modified acid-hydrolyzed method was adopted to extract QT from plasma as follows [41]: 200 μL of plasma sample, 50 μL of kaempferol as an internal standard (20.2 μg·mL^−1^), and 200 μL of 25% hydrochloric acid solution were mixed and vortexed for 2 min. Following hydrolysis in water bath at 90 °C for 15 min, 350 μL ethanol was added after cooling and the obtained mixture was vortexed for 2 min and centrifuged at 8000 rpm for 10 min to yield the supernatant. QT content in the supernatant was determined by HPLC as described in Section 2.6.

### 2.12. Data and Statistical Analyses

The DAS 2.0 software was used to calculate the pharmacokinetic parameters. All data are expressed as the mean ± standard deviation (SD). One-way analysis of variance was used to compare the differences among these groups and *p* < 0.05 meant the difference was statistically significant.

## 3. Results and Discussions

### 3.1. Characterization of QT–NC

**Figure 2:**
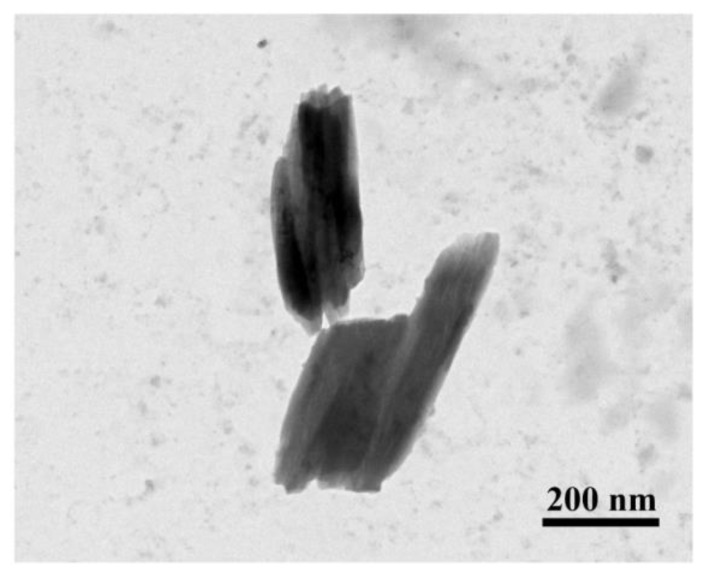
TEM of QT–NC.

The mean size of QT–NC was (354.88 ± 17.35) nm with PDI of 0.106 ± 0.049. Figure 2 showed that QT–NC was a rod in shape with size about 300 to 400 nm.

### 3.2. Optimization of QT–NSSPE Formulation

#### 3.2.1. Fitting the Model to the Data

A total of 17 experiments in a three-factor, three-level Box-Behnken design were required. The independent variables of the 17 runs and the responses that were observed were given in Table 2. The regression analysis was performed using Design Expert software on the data obtained to obtain the Y_1_ and Y_2_ multiple quadratic regression equations. The results of analysis of variance showed that the model F values of Y_1_ and Y_2_ were 18.63 and 11.52 respectively (Table 3), implying the quadratic polynomial models had sufficient statistical significance (*p* < 0.05) to predict the optimal conditions of independent variables. The significance (*p* < 0.05) of each variable was considered to form the quadratic polynomial equation of Y_1_ and Y_2_ as follows:

Y_1_ = 15.45 + 3.32X_1_ − 1.55X_2_ − 1.29X_3_ − 2.64X_1_X_2_ + 3.60X_1_X_3_ − 1.93X_2_X_3_ + 6.33X_1_^2^, (*R*^2^ = 0.9599) (6)

Y_2_ = 4.72 + 0.46X_2_ + 0.27X_3_ + 0.22X_2_X_3_ − 0.30X_1_^2^, (*R*^2^ = 0.9367) (7)

#### 3.2.2. Response Surface Analysis through Polynomial Models

**Figure 3:**
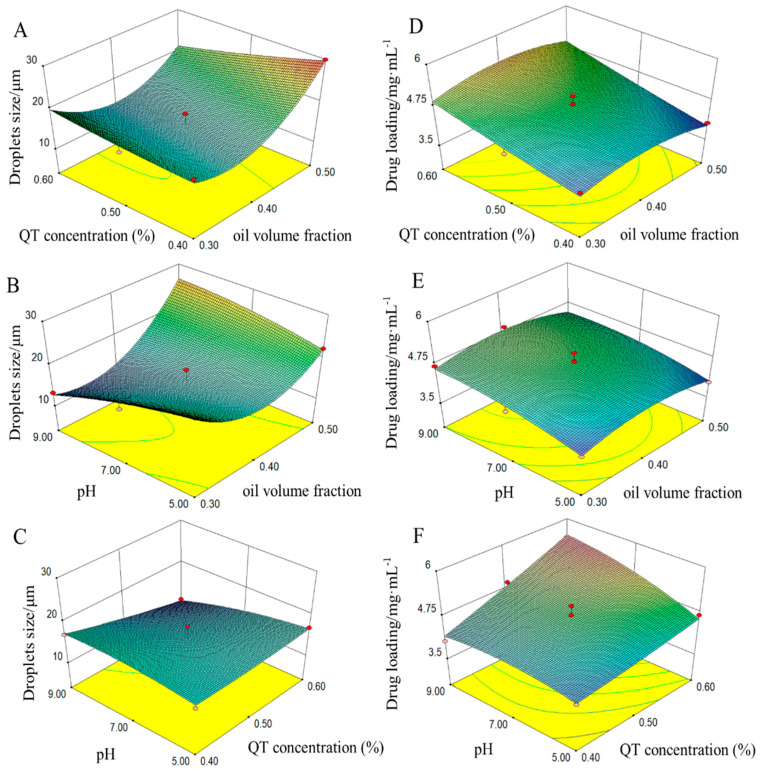
Three-dimensional contour plot showing the effect of independent variables on response of droplets size (Y_1_) and drug loading (Y_2_). (**A**) X_1_ and X_2_ on response Y_1_, (**B**) X_1_ and X_3_ on response Y_1_, (**C**) X_2_ and X_3_ on response Y_1_, (**D**) X_1_ and X_2_ on response Y_2_, (**E**) X_1_ and X_3_ on response Y_2_, (**F**) X_2_ and X_3_ on response Y_2_.

The types of interaction among the three tested variables and the relationship between responses and experimental levels of each variable can be illustrated in 3D response-surface plots. Figure 3A–C was the response surface plots that showed the effect of different independent variables on the droplets size of the QT–NSSPE. The effect of varying QT concentration and oil volume fraction on the droplets size (Y_1_) was studied when the pH was kept constant. As shown in Figure 3A, the changing tendencies of droplets size of the QT–NSSPE that firstly descended and then ascended were observed with the increase of oil volume fractions. Besides, with increase of the QT concentration, the droplets size of QT–NSSPE was decreased. The possible reason is that appropriately reducing the oil volume or increasing the particles concentration could increase the amount of solid particles adsorbed at the oil-water interface thus reducing the free energy of the system, which is helpful to the reduction of droplets size of Pickering emulsions and make it a better stability [42,43]. On the contrary, as the oil volume increases or the particle concentration decreases, a large number of emulsion droplets cannot be completely encapsulated, which will lead to an increase in the droplets size and a decrease in stability of Pickering emulsions. Furthermore, the effect of pH on the droplets size (Y_1_) was studied while the QT concentration was kept constant. As shown in Figure 3B, with the increase of the oil volume fraction while keeping the pH level constant, the droplets size decreased in first and then increased. Because there are increasing in the solubility of the flavonoids as increasing of pH, which may increase the proportion of surface active flavonoid molecules at higher flavonoid concentrations thus enhancing the emulsifying ability of the flavonoid [44,45]. However, at a very high oil volume fractions, the surface of the QT–NSSPE droplets could not be completely coated by the stabilizers. At the same time, enhancing the pH could lead to an increase of the solubility of QT and further reduce the particles in the system, thus increasing the droplets size. Figure 3C illustrated the effect of different QT concentration and pH on the droplets size of QT–NSSPE. As mentioned above, increasing both of them lead to a decrease in the droplets size of QT–NSSPE.

The interaction effects among the three independent variables on drug loading of QT–NSSPE (Y_2_) were studied. As shown in Table 3 and Figure 3D, there were not significant interaction effects between QT concentration and oil volume fractions on the drug loading of QT–NSSPE (*p* = 0.8183), the same phenomenon (Figure 3E) was observed at pH and oil volume fractions (*p* = 0.7020). The reason may be associated with the fact that the oil volume fraction has little influence on drug loading of the QT–NSSPE (X_1_, *p* = 0.8252). The effect of pH and QT concentration on the drug loading (Y_2_) was studied when the oil volume fraction was kept constant. As can be seen in Figure 3F and Table 3, the use of higher pH in combination with the larger QT concentration would lead to an increase in the drug loading of QT–NSSPE. As previously stated, QT has a higher surface active when the pH is relatively alkaline, which is beneficial for the formation of QT–NSSPE. Moreover, the more QT was used in the formulation, the more QT–NC could be adsorbed at the surface of QT–NSSPE droplets, which could result in a higher drug loading of QT–NSSPE.

#### 3.2.3. Optimization and Validation

From this mathematical model, the optimum formulation of QT–NSSPE with the minimum droplets size (Y_1_) and maximum drug loading (Y_2_) was determined by using point prediction of the Design Expert software. Finally, the optimal levels of the three variables were determined as follows: X_1_ (oil volume fraction) = 0.39, X_2_ (QT concentration) = 0.59%, and X_3_ (pH) = 8.92. The corresponding predicted response values were: Y_1_ (size) = 10.16 μm and Y_2_ (drug loading) = 5.49 mg·mL^−1^. Taking into account the reproducibility of the actual operation, X_1_, X_2_, and X_3_ were taken as 0.4, 0.6, and 9, respectively. Three verification experiments were performed using the optimal formulation parameters to validate these predicted values generated according to the results of the Box–Behnken design. Finally, QT–NSSPE with an average of 10.29 μm droplets size and 5.35 mg·mL^−1^ drug loading were obtained, which were in good agreement with the theoretical prediction.

### 3.3. The Morphologies of QT–NSSPE

**Figure 4:**
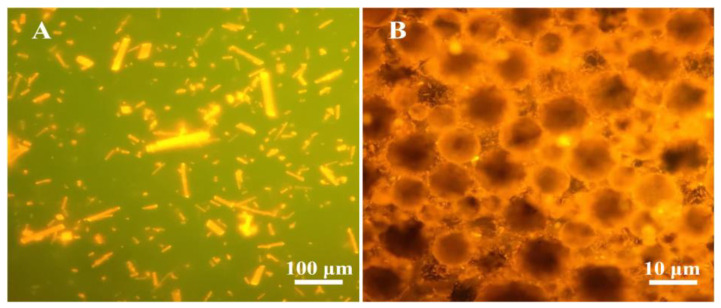
Fluorescence micrographics of (**A**) QT coarse powder, (**B**) QT–NSSPE droplets.

**Figure 5:**
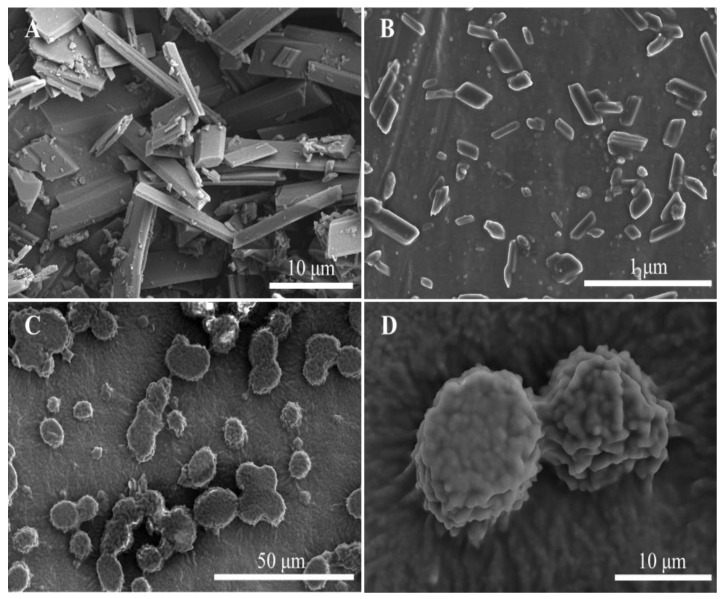
SEM images of (**A**) QT coarse powder (**B**) QT–NC, (**C**) QT–NSSPE, and (**D**) surface of QT–NSSPE droplets.

FM images of QT coarse powder and QT–NSSPE droplets were shown in Figure 4. As can be seen in Figure 4A, strong fluorescence of QT coarse powder was observed, while the equally fluorescence was observed on the surface of QT–NSSPE droplets (Figure 4B), which demonstrated that QT–NC was adsorbed on the surface of QT–NSSPE droplets. In addition, the fluorescence in the interior of emulsion droplets was also observed, indicating the dissolution of quercetin in oil phase. SEM (Figure 5) showed that QT–NSSPE had a dense solid shell formed by QT–NC adsorbed on the surface of emulsion droplets. However, a severe adhesion of emulsion droplets was observed in QT–NSSPE. This may be due to solvent evaporation during the preparation of samples for SEM.

### 3.4. DSC

**Figure 6:**
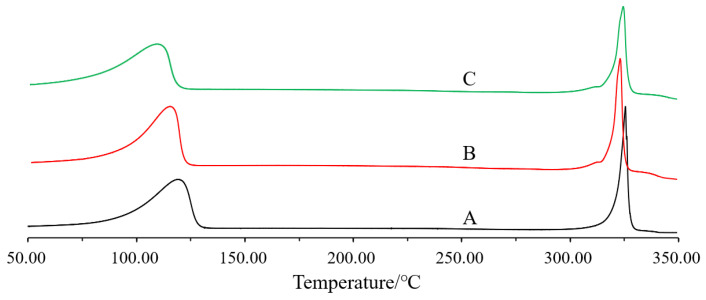
DSC curves of (**A**) QT coarse powder, (**B**) QT–NC lyophilized powder, and (**C**) QT–NC in QT–NSSPE.

The DSC thermograms of QT coarse powder, lyophilized QT–NC, and QT–NC adsorbed in QT–NSSPE were presented in Figure 6. QT coarse powder exhibited a sharp endothermic peak at 321 °C, which was attributed to the melting point of QT [46,47], while the broad endothermic peak between 80 °C and 125 °C was ascribed to the dehydration of QT. The DSC thermogram of QT–NC showed an endothermic peak at 319 °C. An endothermic peak at 321 °C of QT–NC adsorbed in QT–NSSPE was also detected. There were no significantly differences in DSC thermograms of QT in the three samples suggesting the crystalline nature of quercetin has not changed.

### 3.5. XRD

**Figure 7:**
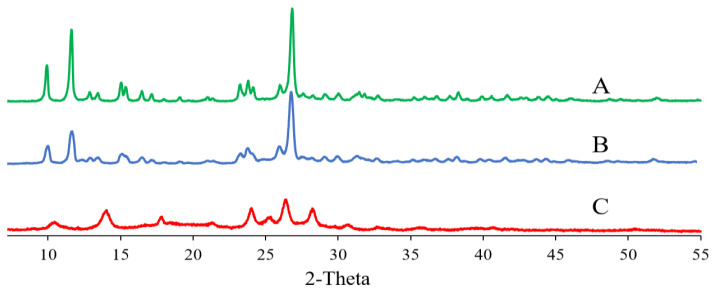
XRD curves of (**A**) QT coarse powder, (**B**) QT–NC lyophilized powder, and (**C**) QT–NC in QT–NSSPE.

XRD was performed to further analyses the crystalline nature of QT coarse powder, lyophilized QT–NC, and QT–NC adsorbed in QT–NSSPE. As shown in Figure 7, QT coarse powder exhibited sharp diffraction peaks at 2*θ* value of 10.8°, 12.4°, and 27.4°, which was in accord with the document [48]. The presence of these sharp diffraction peaks indicated that QT was highly crystalline. Compared with QT coarse powder, the crystalline peaks of lyophilized QT–NC had no significant changes. For QT–NC in QT–NSSPE, the diffraction peaks at 2*θ* value of 10.8° and 27.4° turned into weaker peaks and the diffraction peaks at 2*θ* value of 12.4° has disappeared. The reason may be that the interaction of QT–NC with oil under sonication caused a decrease in crystallinity and a change in crystal nature of QT. There are many factors that induce the decrease of crystallinity or the change of crystal nature, such as temperature, pressure, grinding, and solvent induction [49].

### 3.6. Physical Stability

**Figure 8:**
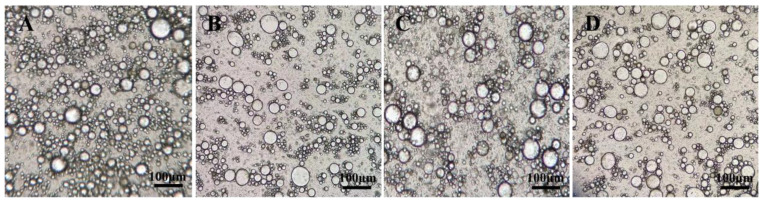
Optical micrographs of QT–NSSPE (**A**) freshly prepared and stored at (**B**) 4, (**C**) 25, and (**D**) 40 °C for 30 days.

**Figure 9:**
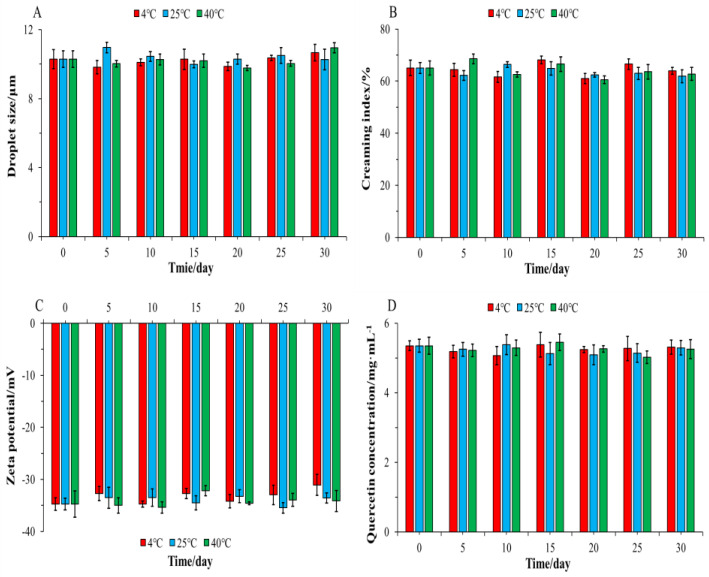
The (**A**) Droplets size, (**B**) CI, (**C**) zeta potential, and (**D**) QT concentration of QT–NSSPE during 30 days storage at 4, 25, and 40 °C. (*n* = 3, mean ± SD).

The stability of QT–NSSPE stored at 4, 25, and 40 °C for 30 days was studied. As shown in Figure 8 and Figure 9, all droplets of QT–NSSPE were spherical before and after 30 days of storage at various situation. The droplets sizes, CI, and zeta potential of QT–NSSPE were very stable with slight change (*p* > 0.05) after storage for 5, 10, 15, 20, 25 and 30 days. The QT concentration of fresh QT–NSSPE was 5.35 mg·mL^−1^, which did not change significantly after 30 days of storage at different temperature (*p* > 0.05). These results indicated that QT–NSSPE had an excellent stability within 30 days of storage at 4, 25, and 40 °C.

### 3.7. In Vitro Drug Release

**Figure 10:**
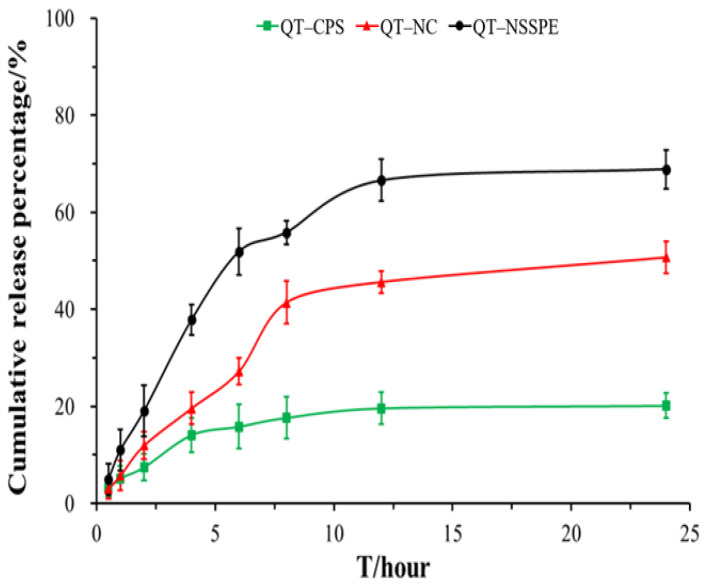
The in vitro dissolution profiles of QT–NSSPE, QT–NC, and QT–CPS in PBS (pH = 7.4) containing 1.0% SDS (1%, *w*/*v*). (*n* = 3, mean ± SD).

Figure 10 showed the dissolution curves of QT–NSSPE, QT–NCS, and QT–CPS in PBS (pH = 7.4) containing SDS (1%, *w*/*v*). QT was released from QT–NC and QT–NSSPE at a much faster speed than from QT–CPS. Within 24 h, 50.71% and 68.88% of QT was dissolved from QT–NC and QT–NSSPE, respectively. However, only 20.15% of QT was released from QT–CPS. Both QT–NC and QT–NSSPE exhibited a significant improvement in drug dissolution as compared to QT–CPS, which may be due to that the form of QT in QT–NC and QT–NSSPE were nanocrystals. The smaller size and larger specific surface area of nanocrystals contribute to a much higher dissolution rate of poorly soluble drugs [50,51]. Compared with QT–NC, QT–NSSPE presented a better dissolution behavior with quicker and more dissolution. This phenomenon was the same as the silybin nanocrystals in self-stabilized Pickering emulsion [37]. A possible reason is that some QT dissolved in the oil phase of QT–NSSPE. The dissolved QT was existed in a form of molecule and had a better release capacity than nanocrystals.

Drug release data of QT–NSSPE were fitted into various kinetics models to elucidate the mechanism and kinetics of drug release (Table 4). According to the value of *R*^2^ for zero-order, first-order, Higuchi, and Korsmeyer-Peppas (0.6328, 0.9905, 0.8539, and 0.8632). QT–NSSPE release profile was best fitted to first-order model, indicating the release mechanism of QT–NSSPE was sustained release. For Korsmeyer-Peppas, the value of ‘*n*’ denotes various mechanisms for the release of drug from the carriers (i.e., Fickian diffusion or non-Fickian diffusion). For *n* ≤ 0.43 corresponds to Fickian diffusion, whereas 0.43 < *n* < 0.85 indicates that diffusion is non-Fickian or anomalous diffusion, and for *n* ≥ 0.85, anomalous diffusion is dominant. In this study, the ‘*n*’ value of Korsmeyer-Peppas was 0.5347, it can be suggested that the mechanism of QT release from QT–NSSPE was anomalous non-Fick diffusion, indicating drug release as a combination of diffusion and erosion of polymer matrix.

### 3.8. Pharmacokinetic Study in Rats

**Figure 11:**
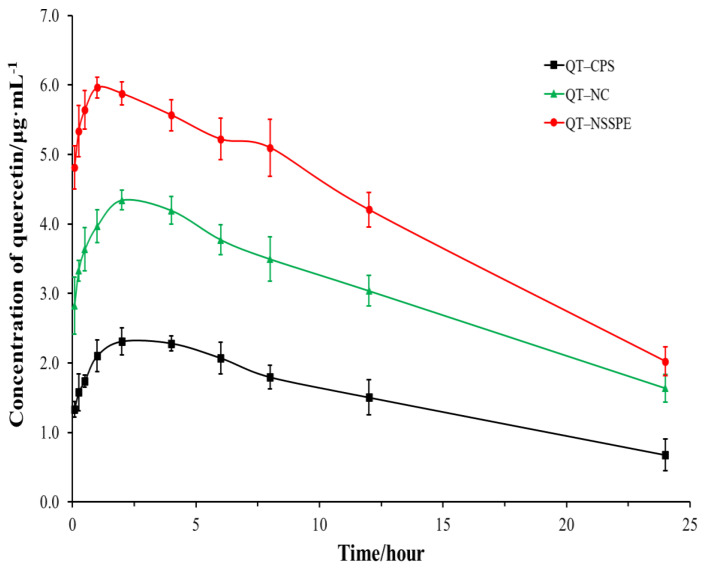
Plasma concentration–time curves of QT–CPS, QT–NC, and QT–NSSPE after a single oral administration at the QT dose of 50 mg·kg^−1^ in rats (*n* = 6, mean ± SD).

The plasma concentration-time profiles and the main pharmacokinetic parameters were presented in Figure 11 and Table 5, respectively. QT–NSSPE and QT–NC exhibited a higher plasma concentration of QT in most of the time point after oral administration compared to those of QT–CPS. As shown in Table 5, after drug administration, the AUC_0–t_ of QT–NSSPE was 2.76 times that of QT–CPS and 1.38 times that of QT–NC (*p* < 0.05). The T_max_ value of QT–NSSPE was (1.75 ± 1.26) h, which was significantly shorter than those of QT–CPS (3.33 ± 1.63) h (*p* < 0.01) and QT–NC (2.96 ± 0.17) h (*p* < 0.05). Furthermore, the C_max_ of QT–NC and QT–NSSPE were 4.43 μg·mL^−1^ and 6.06 μg·mL^−1^, about 1.76 and 2.41 times that of QT–CPS (*p* < 0.05). For t_1/2_ and MRT_0–t_, there were no obvious difference among QT–CPS, QT–NC, and QT–NSSPE (*p* > 0.05).

The above results demonstrated that QT–NSSPE could significantly improve the oral bioavailability of QT, which may be responsible for the follow reasons: (a) Most of QT in QT–NSSPE was in the form of nanocrystals, the smaller size and larger surface area of nanocrystals lead to higher dissolution rate, hence increasing the oral bioavailability of poorly water-soluble drugs [52,53]. (b) With an oily inner core of emulsion droplets, QT–NSSPE had partially characteristic of type I lipid formulations [54]. After oral administration of QT–NSSPE, the oil phase of the emulsion droplets will be digested to form the colloidal species that could interact with endogenous solubilizing species to produce mixed micelles resulting in an enhanced oral absorption of water insoluble drugs [37,55]. (c) Emulsions can also increase drug transportation to the gut-associated lymphoid tissue after oral administration, which promotes the absorption of drugs through the intestinal lymphatics [56]. (d) QT–NSSPE has a higher AUC_0–t_ than QT–NC, because the crystallinity of QT–NC in QT–NSSPE was decreased as proven by XRD. The reduced degree of crystallinity of nanocrystals was responsible, in addition to the nanoparticle size, for the solubility increase [57]. Furthermore, there was part of QT dissolved in the oily inner core of QT–NSSPE droplets, leading a better dissolution profile with a faster rate and more dissolution than QT–NC as confirmed by in vitro dissolution, thus benefiting the oral absorption.

## 4. Conclusions

Quercetin was categorized as the BCS II drug because of its low solubility, which results in a low AUC of QT–CPS and thus limiting its application. There are various methods used to improve the solubility of quercetin, but these methods still have some limitations such as poor stability and large amounts of surfactants. In this study, a new drug delivery system of quercetin, QT–NSSPE, was developed using an ultrasound method. Compared with conventional surfactant emulsions and Pickering emulsions, QT–NSSPE avoids the adverse reactions caused by surfactants and other particles stabilizer. An oil-in-water Pickering emulsion of quercetin (QT–NSSPE) with size of 10.29 μm was developed and optimized by a 3-factor, 3-level Box-Behnken design. In QT–NSSPE, QT–NC acted as solid particle stabilizers as well as therapeutic ingredient without any other surfactants or particles stabilizers. In addition, with a core-shell structure formed by QT–NC adsorbed on the surface of emulsion droplets, QT–NSSPE has not only a good physical stability for a storage of 30 days, but can also enhance the dissolution rate and oral bioavailability of quercetin. In conclusion, the drug nanocrystals self-stabilized Pickering emulsion (NSSPE) could be a promising oral delivery system for insoluble drugs such as quercetin.

## Figures and Tables

**Table 1:** Variables used in Box-Behnken design.

	Level		
	Low (−1)	Medium (0)	High (+1)
Independent variables			
X_1_ = oil volume fractions	0.3	0.4	0.5
X_2_ = QT concentration (%, *w*/*v*)	0.4	0.5	0.6
X_3_ = pH	5.0	7.0	9.0
Dependent variables		Constraints	
Y_1_ = droplets size (μm)		Minimize	
Y_2_ = drug loading (mg·mL^−1^)		Maximize	

**Table 2:** Experimental arrangement with response of Box-Behnken design.

Run	Independent Variables			Response Value	
	X_1_	X_2_ (%, *w*/*v*)	X_3_	Y_1_ (μm)	Y_2_ (mg·mL^−1^)
1	0.40	0.60	9.00	10.59	5.47
2	0.30	0.40	7.00	18.49	4.15
3	0.30	0.50	5.00	22.82	3.96
4	0.40	0.50	7.00	15.09	4.73
5	0.40	0.60	5.00	16.85	4.67
6	0.40	0.40	9.00	16.93	4.03
7	0.40	0.40	5.00	15.07	4.06
8	0.40	0.50	7.00	18.03	4.53
9	0.30	0.50	7.00	16.89	4.27
10	0.50	0.50	5.00	22.22	3.98
11	0.40	0.50	7.00	15.36	4.99
12	0.50	0.40	7.00	29.88	4.03
13	0.50	0.60	7.00	20.27	4.83
14	0.30	0.50	9.00	13.25	4.68
15	0.40	0.50	7.00	14.89	4.72
16	0.40	0.50	9.00	12.25	4.91
17	0.50	0.60	9.00	21.29	5.28

**Table 3:** Analysis of variance (ANOVA) for response surface quadratic mode.

Variables	Y_1_			Y_2_		
	*F*-Value	*p*-Value	Remark	*F*-Value	*p*-Value	Remark
Model	18.630	0.0004	significant	11.520	0.0020	significant
X_1_	32.150	0.0008	**	0.053	0.8252	
X_2_	6.620	0.0368	*	35.670	0.0006	**
X_3_	6.980	0.0333	*	18.300	0.0037	**
X_1_X_2_	8.570	0.0221	*	0.057	0.8183	
X_1_X_3_	21.700	0.0023	*	0.160	0.7020	
X_2_X_3_	8.080	0.0249	*	6.600	0.0371	*
X_1_^2^	76.510	<0.0001	**	10.310	0.0148	*
X_2_^2^	0.170	0.6900		0.073	0.7943	
X_3_^2^	1.320	0.2880		2.770	0.1402	
Lack of Fit	0.850	0.5764	not significant	0.830	0.5847	not significant

* Significant at 5% level, ** Significant at 1% level.

**Table 4:** The fitting results of the release curves of QT–NSSPE.

Model	Equation	*R* ^2^
zero-order	M_t_/M_∞_ = 2.6662t + 20.3551	0.6328
first-order	M_t_ = 71.7454 (1 − e^−0.1904t^)	0.9905
Higuchi	M_t_/M_∞_ = 16.9639t^1/2^ − 0.2666	0.8539
Korsmeyer-Peppas	M_t_/M_∞_ = 19.0519t^0.5347^	0.8632

**Table 5:** Main pharmacokinetic parameters of rats after intragastrical administration of QT–CPS, QT–NC, and QT–NSSPE (*n* = 6, mean ± SD).

Parameter	QT–CPS	QT–NC	QT–NSSPE
AUC_0–t_/μg·mL^−1^·h	35.92 ± 2.42	72.19 ± 6.80 **	99.31 ± 8.39 **^, ##^
MRT_0–t_/h	9.29 ± 0.45	9.78 ± 0.19	9.54 ± 0.13
T_max_/h	3.33 ± 1.63	2.92 ± 0.17	1.75 ± 1.26 **^, #^
C_max_/μg·mL^−1^	2.51 ± 0.23	4.43 ± 0.18 **	6.06 ± 0.38 **^, ##^
t_1/2_/h	12.57 ± 3.72	15.43 ± 3.26	12.97 ± 1.91

** *p* < 0.01 vs. the QT–CPS group. ^#^ *p* < 0.05, ^##^ *p* < 0.01 vs. the QT–NC group.

## Data Availability

All data available are reported in the article.

## Abbreviations

QT: quercetin; NSSPE, drug nanocrystals self-stabilized Pickering emulsions; QT–NC, quercetin nanocrystals; CI, creaming index; FM, Fluorescence Microscope; SEM, Scanning electron microscopy; DSC, Differential Scanning Calorimetry; XRD, X-ray diffraction; QT–CPS, QT coarse powder suspension.

## References

[1] Sun M., Gao Y., Pei Y., Guo C., Li H., Cao F., Yu A., Zhai G. (2010). Development of nanosuspension formulation for oral delivery of quercetin. J. Biomed. Nanotechnol..

[2] Bagheri A., Ebrahimpour S., Nourbakhsh N., Talebi S., Esmaeili A. (2021). Protective effect of quercetin on alteration of antioxidant genes expression and histological changes in the dental pulp of the streptozotocin-diabetic rats. Arch. Oral Biol..

[3] Costa L.G., Garrick J.M., Roquè P.J., Pellacani C. (2016). Mechanisms of Neuroprotection by Quercetin: Counteracting Oxidative Stress and More. Oxid. Med. Cell Longev..

[4] Marefati N., Ghorani V., Shakeri F., Boskabady M., Kianian F., Rezaee R., Boskabady M.H. (2021). A review of anti-inflammatory, antioxidant, and immunomodulatory effects of Allium cepa and its main constituents. Pharm. Biol..

[5] Marunaka Y., Marunaka R., Sun H., Yamamoto T., Kanamura N., Inui T., Taruno A. (2017). Actions of Quercetin, a Polyphenol, on Blood Pressure. Molecules.

[6] Arredondo F., Echeverry C., Abin-Carriquiry J.A., Blasina F., Antúnez K., Jones D.P., Go Y.M., Liang L.Y., Dajas F. (2010). After cellular internalization, quercetin causes Nrf2 nuclear translocation, increases glutathione levels, and prevents neuronal death against an oxidative insult. Free Radic. Biol. Med..

[7] Echeverry C., Arredondo F., Abin-Carriquiry J.A., Midiwo J.O., Ochieng C., Kerubo L., Dajas F. (2010). Pretreatment with natural flavones and neuronal cell survival after oxidative stress: A structure-activity relationship study. J. Agric. Food Chem..

[8] Qi Y., Guo L., Jiang Y., Shi Y., Sui H., Zhao L. (2020). Brain delivery of quercetin -loaded exosomes improved cognitive function in AD mice by inhibiting phosphorylated tau-mediated neurofibrillary tangles. Drug Deliv..

[9] Suganthy N., Devi K.P., Nabavi S.F., Braidy N., Nabavi S.M. (2016). Bioactive effects of quercetin in the central nervous system: Focusing on the mechanisms of actions. Biomed. Pharmacother..

[10] Gao L., Liu G., Wang X., Liu F., Xu Y., Ma J. (2011). Preparation of a chemically stable quercetin formulation using nanosuspension technology. Int. J. Pharm..

[11] Toopkanloo S.P., Tan T.B., Abas F., Alharthi F.A., Nehdi I.A., Tan C.P. (2020). Impact of Quercetin Encapsulation with Added Phytosterols on Bilayer Membrane and Photothermal-Alteration of Novel Mixed Soy Lecithin-Based Liposome. Nanomaterials.

[12] Jaisamut P., Wanna S., Limsuwan S., Chusri S., Wiwattanawongsa K., Wiwattanapatapee R. (2020). Enhanced Oral Bioavailability and Improved Biological Activities of a Quercetin /Resveratrol Combination Using a Liquid Self-Microemulsifying Drug Delivery System. Planta Med..

[13] Ghaffari F., Hajizadeh Moghaddam A., Zare M. (2018). Neuroprotective Effect of Quercetin Nanocrystal in a 6-Hydroxydopamine Model of Parkinson Disease: Biochemical and Behavioral Evidence. Basic Clin. Neurosci..

[14] Shen C., Yang Y., Shen B., Xie Y., Qi J., Dong X., Zhao W., Zhu W., Wu W., Yuan H. (2017). Self-discriminating fluorescent hybrid nanocrystals: Efficient and accurate tracking of translocation via oral delivery. Nanoscale.

[15] Shen B., Shen C., Zhu W., Yuan H. (2021). The contribution of absorption of integral nanocrystals to enhancement of oral bioavailability of quercetin. Acta Pharm. Sin. B.

[16] Heydenreich A.V., Westmeier R., Pedersen N., Poulsen H.S., Kristensen H.G. (2003). Preparation and purification of cationic solid lipid nanospheres--effects on particle size, physical stability and cell toxicity. Int. J. Pharm..

[17] Muller R.H., Keck C.M. (2004). Challenges and solutions for the delivery of biotech drugs—A review of drug nanocrystal technology and lipid nanoparticles. J. Biotechnol..

[18] Tot A., Maksimović I., Putnik-Delić M., Daničić M., Gadžurić S., Bešter-Rogač M., Vraneš M. (2020). The effect of polar head group of dodecyl surfactants on the growth of wheat and cucumber. Chemosphere.

[19] Wang X.Y., Heuzey M.C. (2016). Chitosan-Based Conventional and Pickering Emulsions with Long-Term Stability. Langmuir.

[20] Chuang C.C., Ye A., Anema S.G., Loveday S.M. (2020). Concentrated Pickering emulsions stabilised by hemp globulin-caseinate nanoparticles: Tuning the rheological properties by adjusting the hemp globulin: caseinate ratio. Food Funct..

[21] Tang X.Y., Wang Z.M., Meng H.C., Lin J.W., Guo X.M., Zhang T., Chen H.L., Lei C.Y., Yu S.J. (2021). Robust W/O/W Emulsion Stabilized by Genipin-Cross-Linked Sugar Beet Pectin-Bovine Serum Albumin Nanoparticles: Co-encapsulation of Betanin and Curcumin. J. Agric. Food Chem..

[22] Bhutto R.A., Wang M., Qi Z., Hira N., Jiang J., Zhang H., Lqbal S., Wang J., Stuart M., Guo X. (2021). Pickering Emulsions Based on the pH-Responsive Assembly of Food-Grade Chitosan. ACS Omega.

[23] Marto J., Ascenso A., Gonçalves L.M., Gouveia L.F., Manteigas P., Pinto P., Oliveira E., Almeida A.J., Ribeiro H.M. (2016). Melatonin-based pickering emulsion for skin’s photoprotection. Drug Deliv..

[24] Zhang J., Zhang J., Wang S., Yi T. (2018). Development of an Oral Compound Pickering Emulsion Composed of Nanocrystals of Poorly Soluble Ingredient and Volatile Oils from Traditional Chinese Medicine. Pharmaceutics.

[25] Elmotasem H., Farag H.K., Salama A. (2018). In vitro and *in vivo* evaluation of an oral sustained release hepatoprotective caffeine loaded w/o Pickering emulsion formula-Containing wheat germ oil and stabilized by magnesium oxide nanoparticles. Int. J. Pharm..

[26] Marku D., Wahlgren M., Rayner M., Sjöö M., Timgren A. (2012). Characterization of starch Pickering emulsions for potential applications in topical formulations. Int. J. Pharm..

[27] Sharkawy A., Casimiro F.M., Barreiro M.F., Rodrigues A.E. (2020). Enhancing trans-resveratrol topical delivery and photostability through entrapment in chitosan/gum Arabic Pickering emulsions. Int. J. Biol. Macromol..

[28] Tan H., Zhao L., Tian S., Wen H., Gou X., Ngai T. (2017). Gelatin Particle-Stabilized High-Internal Phase Emulsions for Use in Oral Delivery Systems: Protection Effect and *in Vitro* Digestion Study. J. Agric. Food Chem..

[29] Björkegren S., Nordstierna L., Törncrona A., Palmqvist A. (2017). Hydrophilic and hydrophobic modifications of colloidal silica particles for Pickering emulsions. J. Colloid Interface Sci..

[30] Matos M., Marefati A., Barrero P., Rayner M., Gutiérrez G. (2021). Resveratrol loaded Pickering emulsions stabilized by OSA modified rice starch granules. Food Res. Int..

[31] Sy P.M., Anton N., Idoux-Gillet Y., Dieng S.M., Messaddeq N., Ennahar S., Diarra M., Vandamme T.F. (2018). Pickering nano-emulsion as a nanocarrier for pH-triggered drug release. Int. J. Pharm..

[32] Shahbazi M., Jäger H., Ettelaie R. (2022). A Promising Therapeutic Soy-Based Pickering Emulsion Gel Stabilized by a Multifunctional Microcrystalline Cellulose: Application in 3D Food Printing. J. Agric. Food Chem..

[33] Jiang H., Sheng Y., Ngai T. (2020). Pickering emulsions: Versatility of colloidal particles and recent applications. Curr. Opin. Colloid Interface Sci..

[34] Gambardella C., Morgana S., Bari G.D., Ramoino P., Bramini M., Diaspro A., Falugi C., Faimali M. (2015). Multidisciplinary screening of toxicity induced by silica nanoparticles during sea urchin development. Chemosphere.

[35] Li L., Liu T., Fu C., Tan L., Meng X., Liu H. (2015). Biodistribution, excretion, and toxicity of mesoporous silica nanoparticles after oral administration depend on their shape. Nanomedicine.

[36] Zhang Y., Lu H., Wang B., Wang N., Liu D. (2020). pH-Responsive Non-Pickering Emulsion Stabilized by Dynamic Covalent Bond Surfactants and Nano-SiO Particles. Langmuir.

[37] Yi T., Liu C., Zhang J., Wang F., Wang J., Zhang J. (2017). A new drug nanocrystal self-stabilized Pickering emulsion for oral delivery of silybin. Eur. J. Pharm. Sci..

[38] Wang Z., Hu F., Che Z., Song Q., Shen B.D., Yuan H.L. (2021). Preparation and in vitro release study of quercetin nanocrystals self-stabilized Pickering emulsion. China J. Chin. Mater. Med..

[39] Nifli A.P., Theodoropoulos P.A., Munier S., Castagnino C., Roussakis E., Katerinopoulos H.E., Vercauteren J., Castanas E. (2007). Quercetin Exhibits a Specific Fluorescence in Cellular Milieu:  A Valuable Tool for the Study of Its Intracellular Distribution. J. Agric. Food Chem..

[40] Xu Q., Zhou A., Wu H., Bi Y. (2019). Development and *in vivo* evaluation of baicalin-loaded W/O nanoemulsion for lymphatic absorption. Pharm. Dev. Technol..

[41] Shen B., Wu N., Shen C., Zhang F., Wu Y., Xu P., Zhang L., Wu W., Lu Y., Han J. (2016). Hyperoside nanocrystals for HBV treatment: Process optimization, *in vitro* and in *vivo* evaluation. Drug Dev. Ind. Pharm..

[42] Lim H., Jo M., Ban C., Choi Y.J. (2020). Interfacial and colloidal characterization of oil-in-water emulsions stabilized by interface-tunable solid lipid nanoparticles. Food Chem..

[43] He Y., Wu F., Sun X., Li R., Guo Y., Li C., Zhang L., Xing F., Wang W., Gao J. (2013). Factors that affect Pickering emulsions stabilized by graphene oxide. ACS Appl. Mater. Interfaces.

[44] Luo Z., Murray B.S., Ross A.L., Povey M.J., Morgan M.R., Day A.J. (2012). Effects of pH on the ability of flavonoids to act as Pickering emulsion stabilizers. Colloids Surf. B Biointerfaces.

[45] Wiącek A.E., Chibowski E. (2002). Zeta Potential and Droplet Size of N-tetradecane/ethanol (protein) Emulsions. Colloids Surf. B Biointerfaces.

[46] Chaudhari V.S., Malakar T.K., Murty U.S., Banerjee S. (2021). Extruded filaments derived 3D printed medicated skin patch to mitigate destructive pulmonary tuberculosis: Design to delivery. Expert Opin. Drug Deliv..

[47] Stoyanova N., Spasova M., Manolova N., Rashkov I., Georgieva A., Toshkova R. (2020). Antioxidant and Antitumor Activities of Novel Quercetin -Loaded Electrospun Cellulose Acetate/Polyethylene Glycol Fibrous Materials. Antioxidants.

[48] Ao F., Shen W., Ge X., Wang L., Ning Y., Ren H., Fan G., Huang M. (2020). Effects of the crystallinity on quercetin loaded the Eudragit L-100 electrospun nanofibers. Colloids Surf. B Biointerfaces.

[49] Zhang Q., Mei X.F. (2015). Polymorph transformation of solid drugs. Acta Pharm. Sin..

[50] Lu X., Zhang H., Zheng T., Liu Q., Zhu J., Huang Q. (2020). Evaluation of Oral Bioaccessibility of Aged Citrus Peel Extracts Encapsulated in Different Lipid-Based Systems: A Comparison Study Using Different in Vitro Digestion Models. J. Agric. Food Chem..

[51] Möschwitzer J.P. (2013). Drug nanocrystals in the commercial pharmaceutical development process. Int. J. Pharm..

[52] Gao X., Jasti B.R., Huang M., Wang X., Mahalingam R., Li X. (2020). Design and preparation of nanostructures based on Krafft point of nonionic amphiphiles for delivery of poorly water-soluble compounds. Int. J. Pharm..

[53] Obinu A., Porcu E.P., Piras S., Ibba R., Carta A., Molicotti P., Migheli R., Dalpiaz A., Ferraro L., Rassu G. (2020). Solid Lipid Nanoparticles as Formulative Strategy to Increase Oral Permeation of a Molecule Active in Multidrug-Resistant Tuberculosis Management. Pharmaceutics.

[54] Pouton C.W. (2006). Formulation of poorly water-soluble drugs for oral administration: Physicochemical and physiological issues and the lipid formulation classification system. Eur. J. Pharm. Sci..

[55] Liu Y., Yi T., Huan D., He J.K. (2010). Use of an in vitro lipolysis model to evaluate type I lipid formulations. Acta Pharm. Sin..

[56] Xu H., Gao Y.X., Wang X.T. (2019). Preparation, characterization, and anti-4T1-tumor efficacy of quercetin nanoparticles. Chin. Herbal Med..

[57] Colombo M., Orthmann S., Bellini M., Staufenbiel S., Bodmeier R. (2017). Influence of Drug Brittleness, Nanomilling Time, and Freeze-Drying on the Crystallinity of Poorly Water-Soluble Drugs and Its Implications for Solubility Enhancement. AAPS PharmSciTech.

